# Upconversion nanoparticle platform for efficient dendritic cell antigen delivery and simultaneous tracking

**DOI:** 10.1007/s00604-022-05441-z

**Published:** 2022-09-03

**Authors:** Zhenfeng Yu, Olena Vepris, Christina Eich, Yansong Feng, Ivo Que, Marcel G. M. Camps, Hong Zhang, Ferry A. Ossendorp, Luis J. Cruz

**Affiliations:** 1 Translational Nanobiomaterials and Imaging Group, Department of Radiology, Leiden University Medical Center, Albinusdreef 2, 2333 ZA Leiden, the Netherlands; 2 Van ‘t Hoff Institute for Molecular Sciences, University of Amsterdam, Science Park 904, 1098 XH Amsterdam, the Netherlands; 3 State Key Laboratory of Explosion Science and Technology, School of Mechatronical Engineering, Beijing Institute of Technology, Beijing, 100081 China; 4 Department of Immunology, Leiden University Medical Center, Albinusdreef 2, 2333 ZA Leiden, the Netherlands

**Keywords:** Dendritic cells, Immunotherapy, Upconversion nanoparticles, Pam3CSK4, OVA, *In vivo* monitoring

## Abstract

Upconversion nanoparticles (UCNPs) represent a group of NPs that can convert near-infrared (NIR) light into ultraviolet and visible light, thus possess deep tissue penetration power with less background fluorescence noise interference, and do not induce damage to biological tissues. Due to their unique optical properties and possibility for surface modification, UCNPs can be exploited for concomitant antigen delivery into dendritic cells (DCs) and monitoring by molecular imaging. In this study, we focus on the development of a nano-delivery platform targeting DCs for immunotherapy and simultaneous imaging. OVA 254–267 (OVA24) peptide antigen, harboring a CD8 T cell epitope, and Pam3CysSerLys4 (Pam3CSK4) adjuvant were chemically linked to the surface of UCNPs by amide condensation to stimulate DC maturation and antigen presentation. The OVA24-Pam3CSK4-UCNPs were thoroughly characterized and showed a homogeneous morphology and surface electronegativity, which promoted a good dispersion of the NPs. *In vitro* experiments demonstrated that OVA24-Pam3CSK4-UCNPs induced a strong immune response, including DC maturation, T cell activation, and proliferation, as well as interferon gamma (IFN-γ) production. *In vivo*, highly sensitive upconversion luminescence (UCL) imaging of OVA24-Pam3CSK4-UCNPs allowed tracking of UCNPs from the periphery to lymph nodes. In summary, OVA24-Pam3CSK4-UCNPs represent an effective tool for DC-based immunotherapy.

**Graphical abstract:**

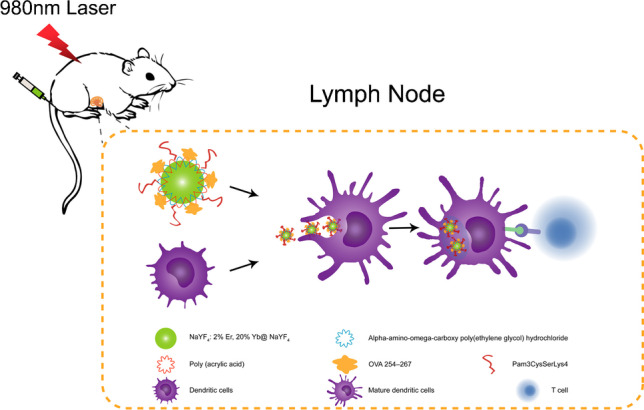

**Supplementary Information:**

The online version contains supplementary material available at 10.1007/s00604-022-05441-z.

## Introduction

Immunotherapy is a treatment that utilizes the cellular immune response to target and kill cancer cells. Antigen-presenting cells (APCs) are vital for effective adaptive anti-tumor immune responses. They express major histocompatibility complex (MHC) class I and MHC class II, costimulatory and various adhesion molecules, thereby enabling them to process and present antigen to T cells in the form of peptide-MHC complexes to initiate both cytotoxic and helper T cell responses. Dendritic cells (DCs), derived from bone marrow precursor cells, are the most powerful and important APCs in our body, and they play a key role in inducing and regulating immune responses [1–3]. DCs can efficiently take up exogenous antigens and present it not only in MHC class II, but also in MHC class I molecules, a process known as cross-presentation. In this way, DCs are able to present exogenously derived peptides to both CD4^+^ T cells and CD8^+^ T cells.

After antigen ingestion, DCs become activated and migrate to the nearest lymph node through the lymphatic system and present the processed antigen to naive T cells and stimulate the proliferation of antigen-specific T cells [4]. Efficient and sustained activation and antigen presentation by DCs in the course of immunotherapy are the major challenges in current immunotherapy approaches.

Although DC-based immune vaccines have been developed, under physiological conditions, DCs may not take up sufficient antigen to elicit immune activation and might require additional stimulation to mature and initiate an immune response [5, 6]. Therefore, a suitable adjuvant is a basic requirement for enhancing the effectiveness of immune vaccines. Toll-like receptors (TLRs) and C-type lectin receptors (CLRs) work in concert to balance immune tolerance and activation. As a pattern recognition receptor on immune cells, including DCs, TLRs can recognize microbial and viral molecular patterns and activate different signal transduction pathways. TLR ligands serve as important immunostimulatory adjuvants for the development of cancer vaccines [7–10]. Pam3CysSerLys4 (Pam3CSK4) is a synthetic triacylated lipopeptide (LP) and a potent TLR2/TLR1 agonist, leading to activation of the pro-inflammatory transcription factor NF-κB [11]. Upon binding to TLR2 on DCs, it activates the downstream signaling cascades that trigger DC maturation, resulting in increased efficiency of antigen processing and presentation [12]. Thus, Pam3CSK4 is considered to be a potential cancer vaccine adjuvant.

In recent years, due to their diverse and flexible characteristics, nanomaterials have been formulated to actively or passively target DCs and to modulate the process of antigen presentation, and are considered to be a powerful tool for constructing DC-targeted vaccines [13–15]. Currently described DC vaccines mainly use lipid and polymer NPs to deliver antigens to DCs and to induce an adaptive immune response [16–20]. DC migration plays an important role in the success of DC immunotherapy [21]. Therefore, non-invasive real-time tracking of DC migration to the lymph nodes could be employed as predictive factor for immunotherapy treatment success. Optical nanoprobes, such as nanoparticles (NPs), are widely used in monitoring and treatment of diseases due to their non-invasiveness and real-time imaging properties, especially in cancer diagnosis and treatment [22, 23]. According to their composition, they can be divided into metallic, organic, inorganic, and polymeric nanostructures [24]. NPs can be utilized as delivery systems for different types of biomolecules, such as fluorophores, metals, peptides, proteins, oligonucleotides, or biomimetic drugs, which give NPs a multifunctional role in diagnosis and treatment [25]. Upconversion NPs (UCNPs) are small NPs (diameter 1–100 nm) that exhibit photon upconversion, a process where two or more photons of relatively low energy are absorbed and converted into one emitted photon with higher energy. This anti-Stokes emission phenomenon of lanthanide-based UCNPs can convert two or more low-energy photons into one high-energy photon to achieve near-infrared to visible (VIS) and even ultraviolet (UV) spectral range. This reaction can effectively avoid the light scattering effect of biological tissues and increase tissue penetration [26, 27]. Therefore, the optical properties of UCNPs combined with surface modification chemistry to link antigen and/or adjuvants for immunotherapy may represent a promising and feasible tool in the field of cancer vaccine.

**Scheme 1:**
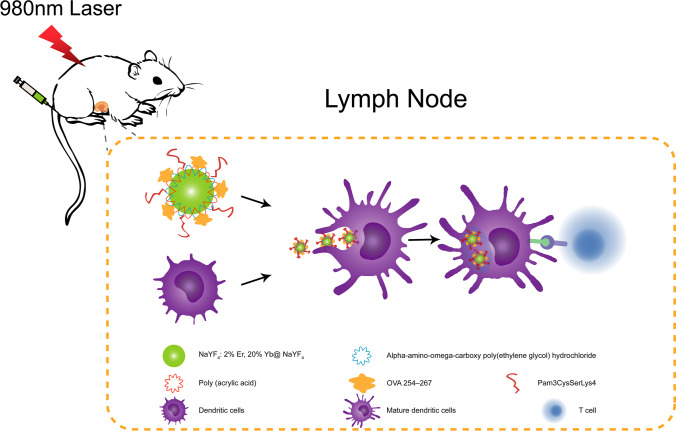
Schematic illustration of antigen-loaded UCNP for DC stimulation, tracking, and vaccination in DC-based immunotherapy

In this work, we have developed an UCNP platform for concomitant antigen and adjuvant delivery to DCs for immunotherapy and simultaneous tracking through *in vivo* upconversion luminescence (UCL) imaging (Scheme 1). Firstly, we synthesized a core–shell structure UCNP with strong luminescence efficiency. Secondly, the UCNP were modified with polyacrylic acid (PAA) to obtain UCNP/PAA-COOH. Finally, the antigen delivery nanoplatform was constructed by sequentially linking alpha-amino-omega-carboxy poly(ethylene glycol) hydrochloride (H_2_N-PEG-COOH), OVA 254–267 (OVA24), and Pam3CSK4 to UCNP/PAA/PEG-COOH via an amide condensation reaction.

H_2_N-PEG-COOH was used to increase the *in vivo* circulation time of the UCNPs. OVA24 (DEVSGLEQLESIINFEKLAAAAAK), a 24-residue long synthetic antigenic peptide harboring a cytotoxic T-lymphocyte (CTL) epitope of ovalbumin (SIINFEKL), which can be recognized by CD8^+^ T cells derived from OT-1 TCR transgenic mice, served as a model antigen to quantify cytolytic T cell responses [28]. Our results show that UCNP/PAA/PEG/OVA24/Pam3CSK4 could enhance DC activation, antigen presentation, and T cell activation *in vitro* and *in vivo*. In addition, UCL imaging could be used to monitor UCNP trafficking from the injection site to the draining lymph nodes after intradermal injection. This work provides a new strategy for the development of an UCNP-based nanovaccine.

## Materials and methods

ReCl_3_·6H_2_O (Er, Yb, Y > 99%), oleic acid (OA), oleylamine (OM), 1-octadecene (ODE), yttrium trifluoroacetate ((CF_3_COO)_3_Y), sodium trifluoroacetate (CF_3_COONa), sodium hydroxide (NaOH), ammonium fluoride (NH_4_F), N-hydroxy-succinimide (NHS), 1-ethyl-3-(3-dimethylaminopropyl) carbodiimide (EDC), fluorescein isothiocyanate (FITC), methanol, ethanol, acetone, cyclohexane, N,N-dimethylformamide (DMF), dimethyl sulfoxide (DMSO), and polyacrylic acid (PAA) were purchased from Sigma-Aldrich. All chemicals were used without further purification. DAPI and anti-CD86-FITC (clone GL1) were purchased from Thermo Fisher. Anti-CD69-PE (clone H1.2F3), anti-CD62L-APC (clone MEL-14), and anti-CD3 (clone 145-2C11) were purchased from BioLegend. Alpha-Amino-omega-carboxy poly(ethylene glycol) hydrochloride (H_2_N-PEG-COOH*HCL, MW 3.000 Dalton) was purchased from Iris Biotech. Cell Titer 96 AQueous MTS Reagent Powder was purchased from Promega. Anti-CD45.1 (clone A20), CD8α + (clone 53–6,7), and anti-CD43 (clone 1B11) were purchased from BioLegend.

### Synthesis of NaYF_4_: 2% Er, 20% Yb@ NaYF_4_ nanoparticles (UCNP)

UCNPs were synthesized by adding (CF_3_ COO)_3_Y and CF_3_COONa to a mixed solution of OA (6 mL), OM (6 mL), and ODE (10 mL), followed by stirring at 65 °C for 30 min under nitrogen. Then, the mixture was heated to 290 °C and left to react for 1 h. After the reaction, the obtained UCNP precursor was centrifuged and washed with acetone and ethanol, and then dispersed in ODE.

In a typical experiment, ODE and OA were added to 1 mmol ReCl_3_·6H_2_O. After thorough stirring, the solution was heated to 160 °C under nitrogen. NaOH and NH_4_F in methanol were added dropwise, and then heated to 75 °C to remove methanol. The mixture was left to react under atmospheric nitrogen at 300 °C for 1 h, and then injected into the previously prepared NaYF_4_ solution. The reaction was left to react for another 30 min. The mixture was centrifuged and washed with acetone and ethanol, and then dispersed in cyclohexane.

### Surface functionalization of UCNP

The COOH-functionalized UCNP were synthesized via a ligand exchange to phase transfer following a reported method [29]. Briefly, the synthesized UCNPs were dispersed in cyclohexane and reacted with hydrochloric acid for 4 h, and washed with water. Then a DMF solution containing PAA was added, and the mixture was allowed to react for 24 h under stirring. The NPs were washed with water and DMF, and dispersed in DMF.

### Conjugation of H_2_N-PEG-COOH/OVA24/Pam3CSK4 to UCNP/PAA-COOH

First, the surface of UCNP/PAA-COOH was modified with H_2_N-PEG-COOH via a crosslinking reaction where a water soluble carbodiimide was used to activate a carboxylic group. UCNP/PAA-COOH (30 mg) were dissolved in DMSO, and then EDC (17.4 mg) and NHS (12.89 mg) were added drop by drop to the dissolved acid. The mixture was left stirring for 1 h at room temperature, and then centrifuged and washed with deionized water. Subsequently, H_2_N-PEG-COOH was added to the solution, stirred overnight, centrifuged, and washed in D.I. water.

The method of conjugating OVA24 and Pam3CSK4 was similar as above. Firstly, a mixture of EDC and NHS (EDC: NHS = 1:1.1) was added dropwise to UCNP/PAA/PEG-COOH solution (pH adjusted to 4.4), stirred for 3 h, and then centrifuged to obtain UCNP/PAA/PEG-NHS. Secondly, the resulting material was resuspended in DMF, and OVA24 and Pam3CSK4 were added into the solution, stirred overnight at 4 °C, and then centrifuged to obtain UCNP/PAA/PEG/OVA24/Pam3CSK4.

The size and zeta potential of the UCNPs (1 mg/mL) were measured by the Malvern ZetaSizer 2000 (Malvern, UK), and the morphology was visualized by a Tecnai T12 Transmission Electron Microscope (FEI; Oregon, USA) equipped with an OneView Camera Model 1095 (Gatan; Pleasanton, USA) at a voltage of 120 kV. Samples were prepared by adding 1 mg/mL of UCNP solution to the surface of glow-discharged copper grids. The diameters of the UCNPs in the TEM images were calculated by ImageJ software.

### MTS assay

The cellular toxicity of the different UCNP formulations was analyzed by MTS assay. Briefly, D1 cells (1 × 10^4^) were plated in 96 wells and incubated at 37 °C for 24 h. The cells were then treated with different concentrations of UCNPs (0–200 µg/mL) for 48 h. Afterwards, the medium was removed and 100 µL of fresh medium and 20 μL of MTS reagent were added to each well according to the manufacturer’s instructions. The cells were then incubated in an incubator at 37 °C for 1.5 h. The absorbance (OD) was read at 490 nm using microplate reader (Molecular Devices, USA). Cell viability was analyzed by the ratio to the untreated control group. Data are expressed as mean ± SD.

### Fluorescent microscopy

The uptake and intracellular localization of the different UCNP formulations in D1 cells was determined by fluorescent microscopy (Leica DMRA fluorescence microscopy) equipped with a 980 nm laser (1000 mW/cm^2^). D1 cells were seeded on poly-L-Lysine coated cover slips and incubated overnight with UCNPs. Then, the coverslips were washed with medium and fixed for 10 min at room temperature with 4% paraformaldehyde. Subsequently, the cells were washed, labeled with DAPI for 5 min, and sealed in mounting medium (Mowiol).

### DC maturation assay

- Interleukin 12 (IL-12) production by ELISA

Murine D1 DCs (5 × 10^4^ cells/well) and UCNPs (50 µg/mL) were incubated in a 96-well plate in a 37 °C incubator for 24 h. At the end of the incubation time, the supernatant was collected. Then, flat-bottom NUNC MaxiSorp96-well plates were coated with IL-12 capturing antibody (BioLegend) overnight at 4 °C, followed by several washing steps in sodium chloride/0.05% Tween-20 and a blocking step (in PBS/0.05%, Tween-20/1% BSA) for 1 h at RT. After washing, the plates were incubated with diluted samples (250 ×) and recombinant mouse IL-12 (standard curve) for 1 h at RT. The plates were washed, followed by incubation with IL-12 biotinylated detection antibody (BioLegend) for 1 h at RT followed by several washing steps in sodium chloride/0.05% Tween-20. Then, streptavidin-HRP (BioLegend) was added to each well and incubated for 1 h at RT. After several washing steps, substrate solution (3,3′,5,5′-tetramethylbenzidine (TMB)) (Sigma-Aldrich) was added and incubated for 15–30 min in the dark. At last, H_2_SO_4_ was added to each well to stop the reaction. The plates were immediately measured at 450 nm with an ELISA reader.

(b) Flow cytometry

Briefly, murine D1 DCs and UCNPs were co-cultured in a 96-well plate in a 37 °C incubator for 24 h. The cells were washed with FACS buffer (PBS/0.5% BSA/0.02% sodium azide) and stained with anti-CD86-FITC (Thermo Fisher, USA) antibody. After 30 min, the cells were washed and resuspended in 100 μL FACS buffer. A LSR-II cytometer (BD Biosciences, USA) was used to measure the samples, and the data was analyzed by FlowJo (version 10).

### *In vitro* antigen presentation assay

OT1 cells were isolated from the spleen or/and lymph nodes of OT-1 TCR transgenic mice. *In vitro* cross-presentation of OVA by DCs was studied by incubating titrated amounts of OVA surface-modified and control UCNPs or soluble OVA (matching the amount linked to the UCNPs) with 5 × 10^4^ D1 cells at 37 °C, followed by addition of 5 × 10^4^ OT-1 cells. After incubation for 48 h at 37 °C in a 96-well plate, the cells were centrifuged and washed with PBS/BSA/sodium azide, and labeled with anti-CD69 and anti-CD62L antibody for 20 min on ice in the dark. Then, the cells were washed three times by adding PBS/BSA/sodium azide to each well and centrifuge. Finally, the cells were resuspended in PBS/BSA/sodium azide and measured by flow cytometry. The direct MHC-binding minimal epitope SIINFEKL (100 ng/mL in PBS) was used as a positive control, and unstimulated D1 cells as negative control.

### *In vivo* antigen presentation assay

To establish antigen presentation *in vivo*, C57BL/6 mice were vaccinated with OVA24/Pam3CSK surface-modified UCNPs or with OVA alone in its soluble form. Mice were injected subcutaneously with a 2 mg/mL suspension of UCNPs in PBS containing 28 µg of encapsulated OVA or soluble OVA. One day (d) after vaccination, 5 × 10^5^ OVA-specific OT-1 CD8þ T cells were injected intravenously in the tail vein in 200 µL PBS. Mice were sacrificed 5 d after T cell transfer to analyze the expansion of the transferred OVA-specific T cells caused by presentation of the OVA antigen. Vaccine-draining inguinal lymph nodes and the spleen were dissected, mashed on 70-µm cell strainers (Becton Dickinson), and the cells were stained with fluorescently labeled antibodies for CD3, CD8β, the OT-1 congenic marker CD45.1, CD62L, and CD43, and were analyzed by flow cytometry. The data is presented as the percentage of CD62L^+^ and CD43^+^ T cells within the population of CD45.1^+^CD3^+^CD8α^+^ OT-1 T cells.

### *In vivo* UCL imaging

Albino C57BL/6 mice were vaccinated intradermally (left and right to the tail base) with each 30 µL of a 2 mg/mL suspension of UCNP/PAA/PEG and UCNP/PAA/PEG/OVA24/Pam3CSK dispersed in PBS. The luminescence signal was then studied at several time points after administration by using the IVIS spectrum preclinical *in vivo* imaging system (PerkinElmer, Massachusetts, USA). The interior platform of the animal housing unit of the IVIS Spectrum imager was adapted to hold a clamp which was attached onto a 980-nm laser head (600 mW/cm^2^). The power supply for the laser was placed outside of the imager but connected by wires inserted through the door entrance of the imager. The fluorescent signal was measured by quantifying the fluorescent intensity in pre-set regions of the two vaccine-draining inguinal lymph nodes, expressed as the total radiant efficiency in p/sec/cm^2^/sr. The data is presented in two different ways. Firstly, the fluorescent signal is presented as the percentage of the maximum recorded value, to show percentual decrease in time. Secondly, the data is presented as the signal to background ratio and it is calculated by dividing the fluorescence signal of the lymph nodes by the background signal (after PBS injection) at each time point.

### Statistical analysis

The data were analyzed by GraphPad Prism 8.0.1 software. The data are expressed as mean ± standard deviation (SD). Statistical significance of differences is indicated as *p < 0.05, **p < 0.01, ***p < 0.001, and ****p < 0.0001.

### Ethics approval

The *in vivo* study was performed complying with the Dutch National Law on animal experiments, after the approval of the research protocol by the LUMC Animal Welfare Committee (Register: PE.16.052.286 and PE.16.052.287; AVD116002015271).

## Results and discussion

### Preparation and characterization of NPs

**Fig. 1:**
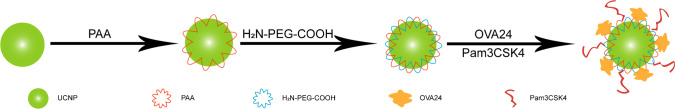
The surface modification process of UCNP/PAA/PEG/OVA24/Pam3CSK4

The NaYF_4_: 2% Er, 20% Yb@ NaYF_4_ UCNPs were prepared according to a reported protocol [29] and further surface-modified as illustrated in Fig. 1. Briefly, after UCNP preparation, the synthesized oleic acid-capped UCNPs were functionalized with PAA to obtain the hydrophilic acid-terminated UCNP (UCNP/PAA-COOH). Subsequently, in order to increase the biocompatibility of the UCNPs, the H_2_N-PEG-COOH was modified on its surface to form UCNP/PAA/PEG-COOH. At last, excess OVA24 and Pam3CSK4 were conjugated to UCNP/PAA/PEG-COOH by an amide condensation reaction to obtain the final UCNP/PAA/PEG/OVA24/Pam3CSK4. The resulting UCNP formulations were characterized by transmission electron microcopy and zeta potential measurements (Table S1).

Transmission electron microcopy analysis of the UCNP/PAA/PEG revealed that the NPs were spherical and monodispersed with an average size of ∼25 nm (50 randomly selected). Zeta potential measurements showed a slightly negative surface charge of − 7 mV. With the gradual functionalization of the UCNP surface, the negative surface charge increased, which indirectly proves that with each addition of substance, the surface of the UCNP was successfully modified.

**Fig. 2:**
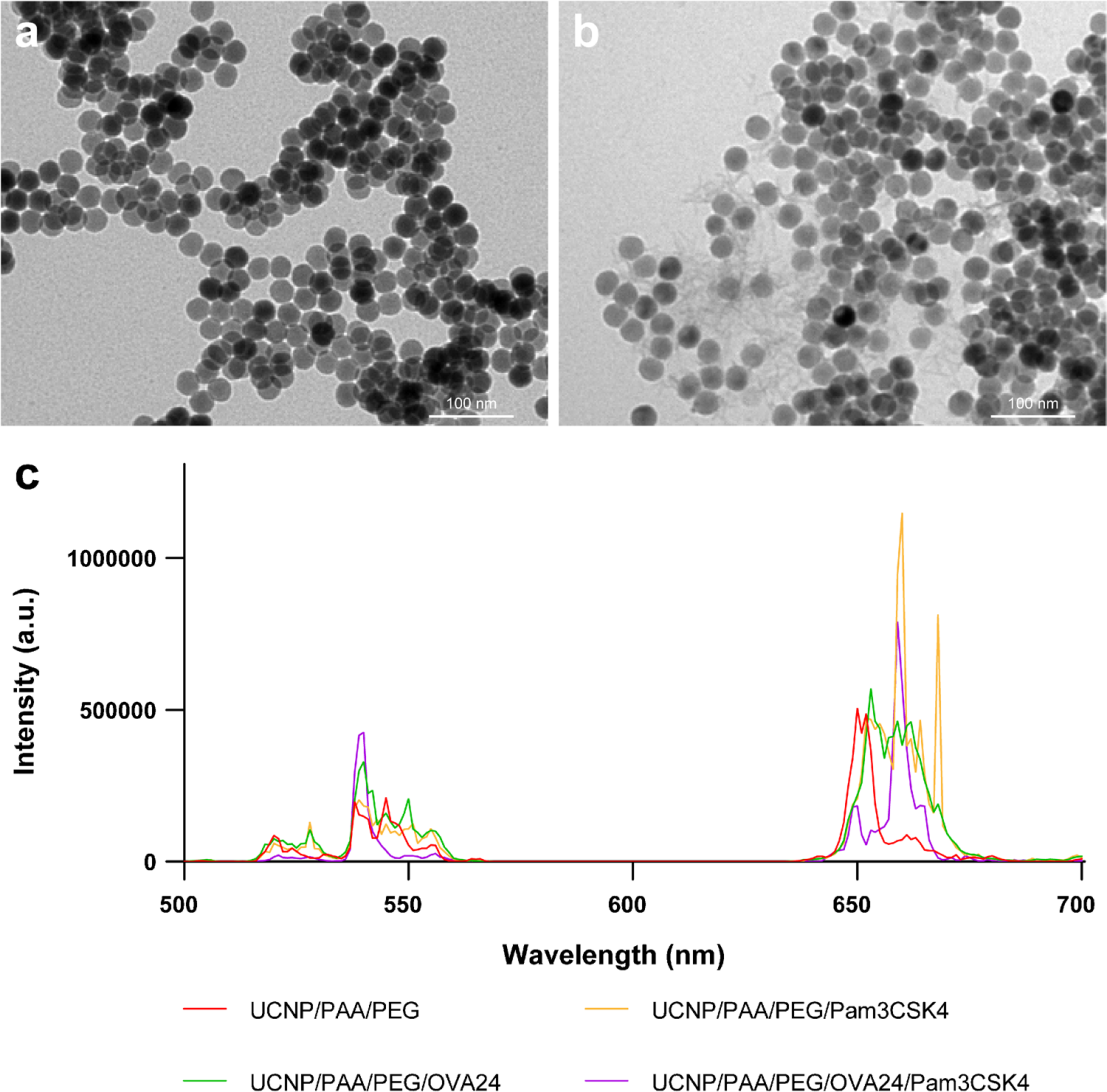
TEM images of UCNP/PAA/PEG (**a**) and UCNP/PAA/PEG/Pam3CSK4 (**b**), scale bar: 100 nm. Upconversion luminescence spectra of polymer-coated UCNP dispersed in water at a concentration of 1 mg/mL and excited with 980-nm laser at laser power of 600 mW/cm.^2^ (**c**)

After surface functionalization, TEM analysis confirmed the presence of Pam3CSK4 attached to the surface of UCNP/PAA/PEG (Fig. 2b). Next, we characterized the optical properties of the UCNP formulations after surface modification. Under 980 nm NIR laser excitation, the UC emissions at 520 nm, 540 nm, 640 nm, and 660 nm were basically consistent with the emission peaks of bare UCNPs. In addition, we found that the samples containing Pam3CSK4 showed stronger UCL emission signal in the far-red region, with a small shift from 640 to 660 nm. In conclusion, this result proves that the polymer and protein coating of the UCNPs did not negatively affect their UCL emission signal. Thus, antigen/adjuvant surface-modified UCNPs are suitable not only as an antigen delivery platform but also as a tracking system.

### *In vitro* cytotoxicity

**Fig. 3:**
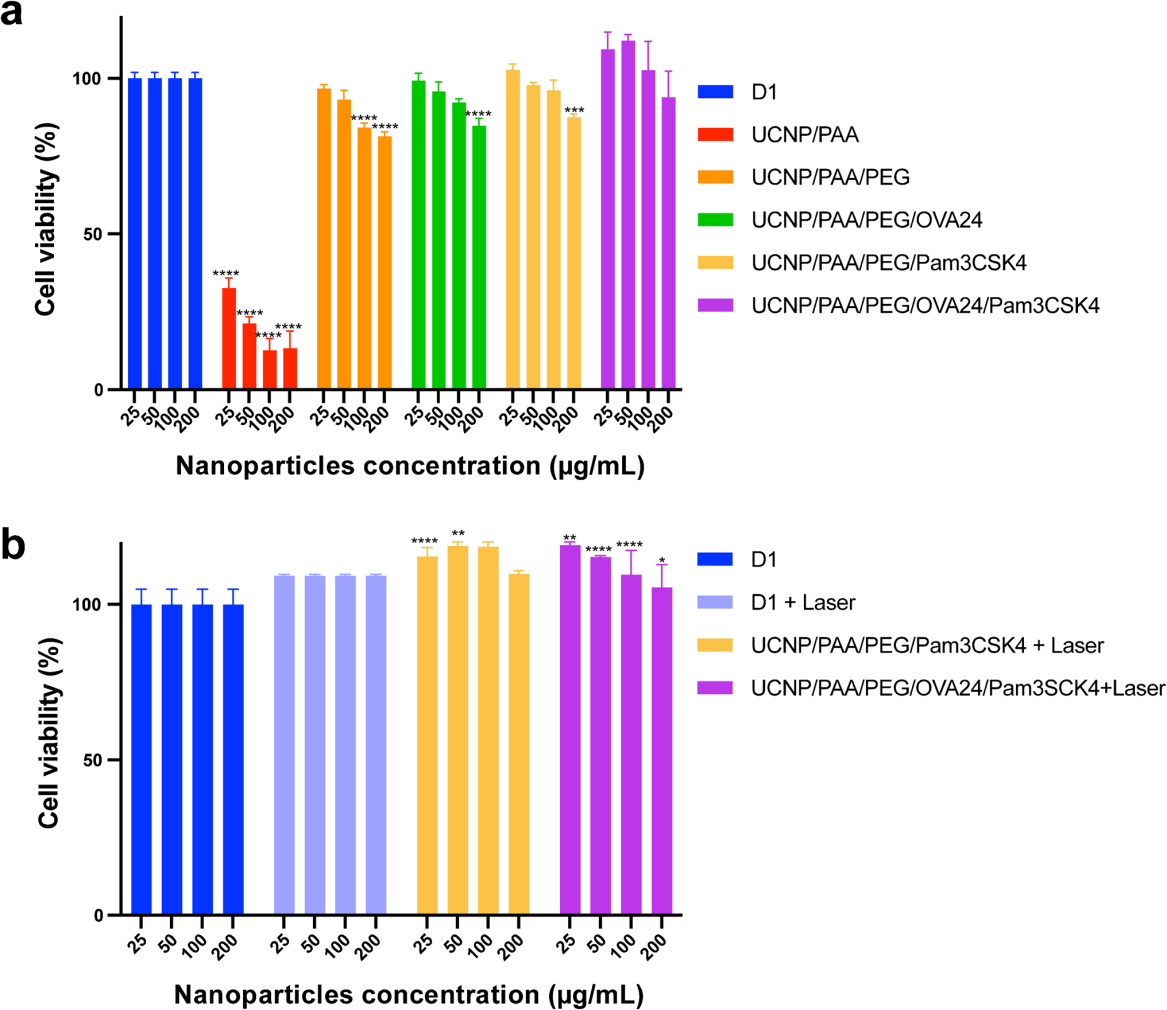
*In vitro* cell viability assay of D1 cells after incubation with UCNPs for 48 h (**a**) and (**b**) treated with 980 nm laser at 600 mW/cm.^2^. Statistical significance was calculated using two-way ANOVA, by comparing experimental groups to the control group (*p < 0.05, **p < 0.01, ***p < 0.001, and ****p < 0.0001)

Next, we investigated whether the UCNPs would have any cytotoxic effect on DCs. To this end, we co-cultured murine DCs (D1 cells) with different concentrations of UCNPs (12.5–200 µg/mL) for 48 h and analyzed the cell viability by MTS assay (Fig. 3a). We found that with increasing concentrations of UCNP/PAA, the viability of DCs was significantly reduced, compared to untreated D1 cells. When the surface of UCNP/PAA was additionally modified with H_2_N-PEG-COOH (UCNP/PAA/PEG), the cytotoxic effect was greatly reduced and only observed at high concentrations of UCNPs of ≥ 100 µg/mL. When the surface of UCNP/PAA/PEG was modified with OVA24, or Pam3CSK4, we observed a further reduction of the cytotoxic effect on DCs. UCNP/PAA/PEG/OVA24/Pam3CSK4 did not induce any cytotoxicity at all. In summary, our findings indicate that surface modification of UCNPs with H_2_N-PEG-COOH, OVA24, and Pam3CSK4 reduced cellular cytotoxicity of UCNP/PAA, possibly by shielding the adenosine triphosphate (ATP) deprivation caused by the strong binding between the lanthanide element ions and the phosphate groups of intracellular ATP, as reported in other UCNP studies, thus effectively reducing the toxic effects of lanthanide ions [30].

Next, we checked whether laser excitation and resulting UCL would have any cytotoxic effect on DCs. To this end, we incubated D1 cells with different concentrations of UCNP/PAA/OVA24/Pam3CSK4 and exposed the cells to 980-nm laser irradiation (Fig. 3b). Forty-eight hours after laser exposure, cell viability was measured by MTS assay. It was found that neither laser exposure nor the resulting UCL of the UCNPs tested at different concentrations (12.5–200 μg/mL) induced cytotoxic effects in D1 cells (Fig. 3b).

### Uptake of UCNP/PAA/PEG/OVA24/Pam3CSK4 by DCs

**Fig. 4:**
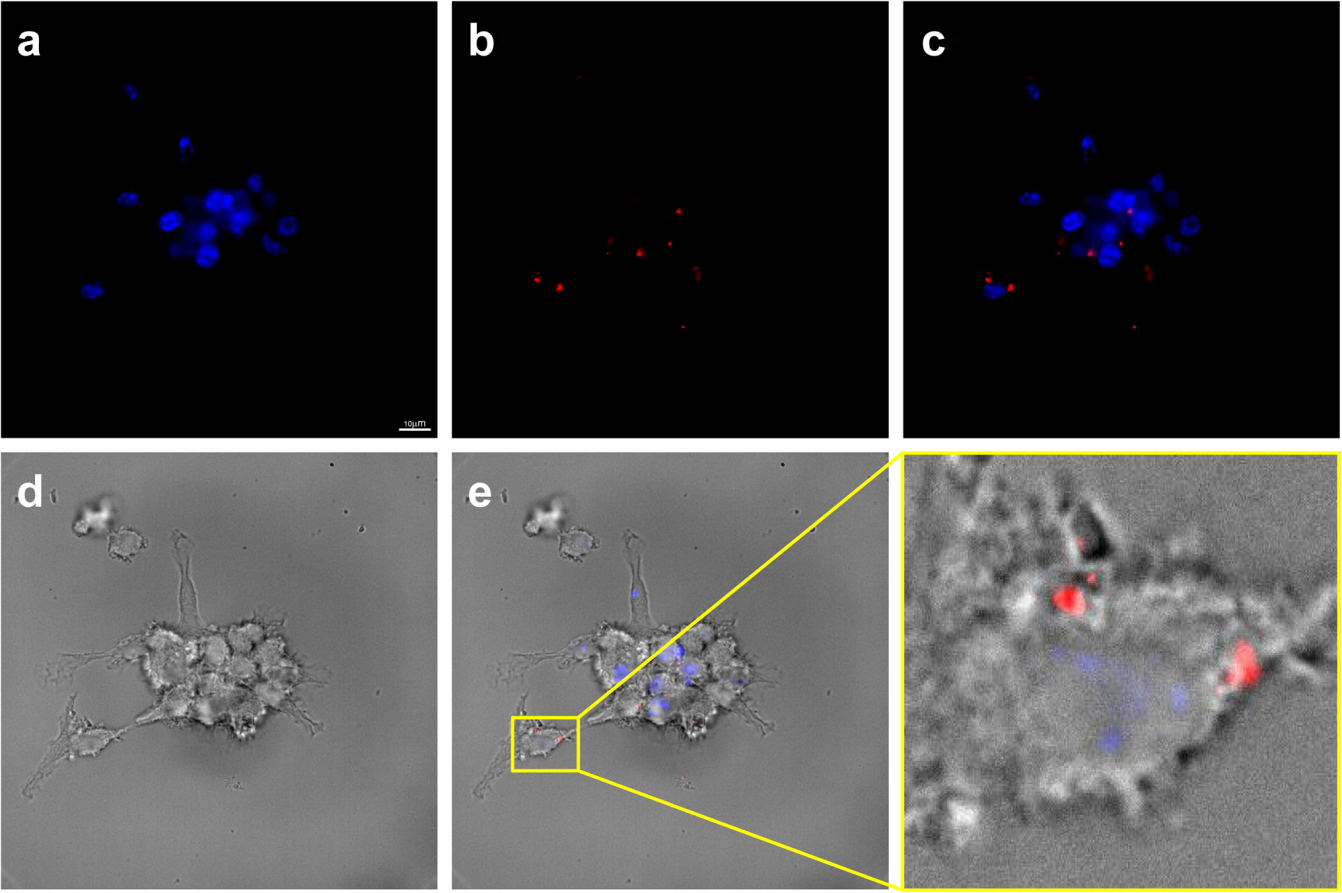
Uptake of UCNP/PAA/PEG/OVA24/Pam3CSK4 by DCs. Confocal microscope images show uptake of functionalized UCNPs after 4 h of incubation at 37 °C by D1 DCs. Uptake was visualized based on UCL properties of NPs composed of NaYF_4_: 2% Er, 20% Yb@ NaYF_4_ (excitation wavelength at 980 nm and emission wavelength at 650 nm). DAPI (**a**), UCNP/PAA/PEG/OVA24/Pam3CSK4 (**b**), overlay DAPI and UCNPs (**c**), brightfield (**d**), composite (**e**). Scale bare: 10 µm

As the purpose of this study was to design an antigen/adjuvant delivery system for DCs, we next wondered how well the UCNPs would be taken up by DCs *in vitro*. To this end, we incubated DCs with UCNP/PAA/PEG/OVA24/Pam3CSK4 for 4 h. After incubation with NPs, the cells were washed and the nucleus was labeled with DAPI. The UCNPs were imaged using a fluorescent microscope equipped with a 980 nm laser line, and the UCL was collected at 660 nm. At 980 nm laser irradiation, the fluorescence microscope image clearly revealed an intracellular UCL signal of UCNP/PAA/PEG/OVA24/Pam3CSK4 (Fig. 4b), providing evidence that DCs efficiently engulfed NPs. Efficient NP uptake is a crucial prerequisite for DCs to process Pam3CSK4 and OVA24 linked to the UCNP surface, and to present antigen to T cells.

### *In vitro* maturation of DCs

**Fig. 5:**
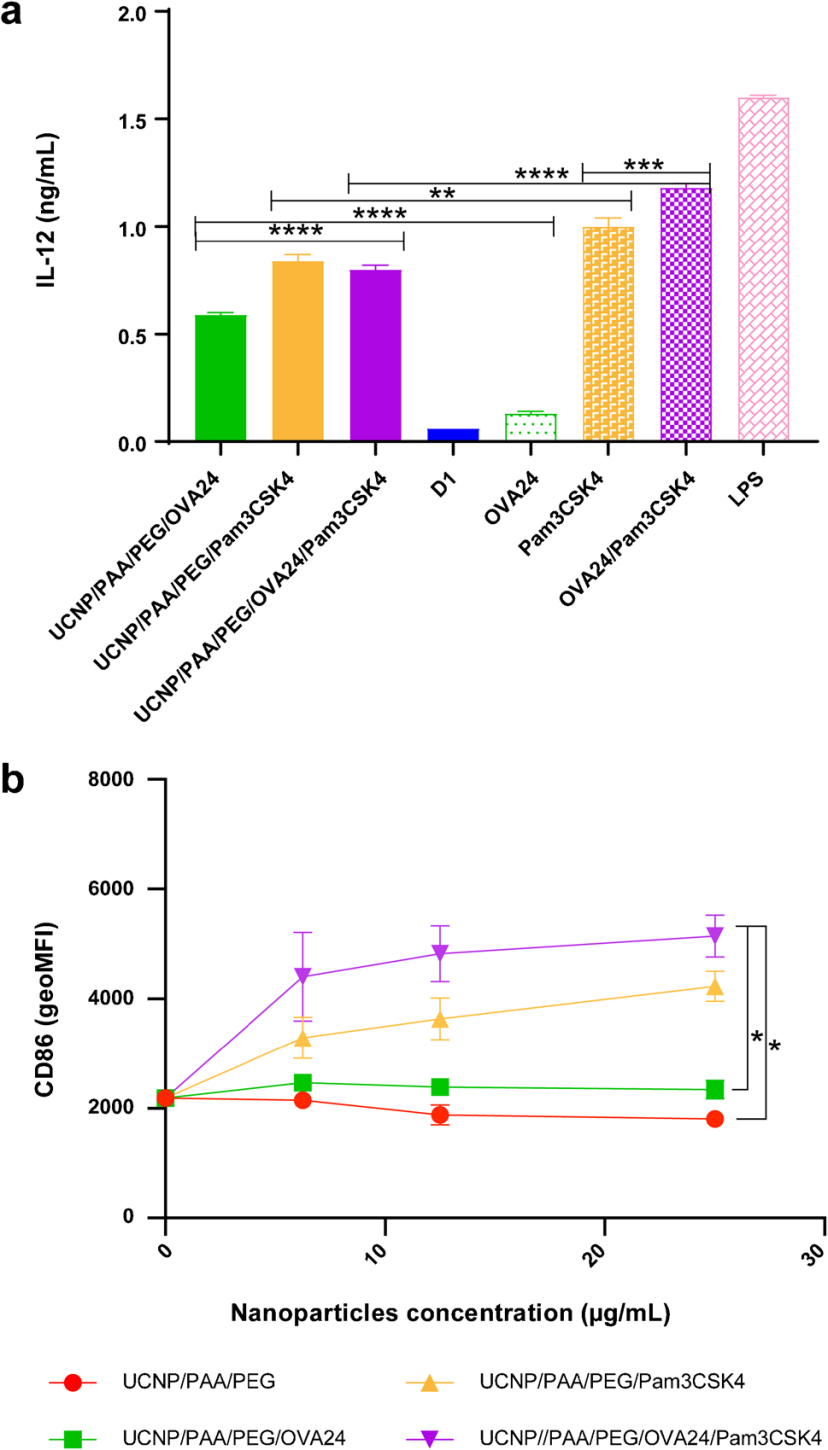
*In vitro* maturation of D1 DCs was assessed after incubation with titrated amount of UCNPs for 24 h. At the end of the incubation time, the supernatant was harvested and the amount of IL-12 was determined by ELISA (**a**). The differences in IL-12 production were estimated applying Tukey’s multiple comparisons test. Shown is one representative experiment and the data are shown as mean ± SD. The D1 cells were stained with anti-mouse CD86 antibody and the geometric mean of the CD86 signal was determined via flow cytometry (**b**). Statistical significance was calculated using one-way ANOVA (*p < 0.05, **p < 0.01, ***p < 0.001, and ****p < 0.0001)

A crucial question is whether surface-conjugated adjuvant and antigen could efficiently stimulate DCs. Thus, in the next step, we assessed the immunostimulatory potential of the different UCNP formulations on the basis of their ability to induce maturation of DCs, and to support antigen presentation upon uptake by DCs. To monitor the ability of the UCNPs to activate DCs, the level of IL-12 production upon co-culture of DCs with UCNPs was measured by enzyme-linked immunosorbent assay (ELISA). IL-12 secretion by DCs is a good indication of the immunostimulatory potential of a compound, since this cytokine is important for the development of T cell-based immune responses [31]. From Fig. 5a, we can see that the addition of soluble Pam3CSK4 and OVA24/Pam3CSK4 stimulated DCs to significantly increase the production of IL-12, compared to unstimulated cells, similar to the levels observed after stimulation with LPS. In contrast, OVA24 alone did not lead to stimulation of IL-12 production by DCs. When DCs were co-cultured with different formulations of UCNPs, UCNP/PAA/PEG/Pam3CSK4, and UCNP/PAA/PEG/OVA24/Pam3CSK4, the production of IL-12 significantly increased, compared to untreated cells. However, we also observed an increase in IL-12 production upon treatment with UCNP/PAA/PEG/OVA24, suggesting that the UCNPs alone might have immunomodulatory properties. Overall, compared with DCs treated with free OVA24, Pam3CSK4, or OVA24/Pam3CSK4, the level of IL-12 secreted by DCs after incubation with UCNP surface-modified with OVA24, Pam3CSK4, or OVA24/Pam3CSK4 was slightly lower. This can be explained by the difference in uptake kinetics of soluble versus NP-delivered substances. When surface-modified, as in the case of UCNP/PAA/PEG/OVA24/Pam3CSK4, steric hindrance might prevent Pam3CSK4 efficiently interacting with its receptor TLR2, while soluble Pam3CSK4 can readily engage in receptor/ligand interactions. In addition, to fully exert its potential as adjuvant, Pam3CSK4 first need to be cleaved from the UCNP backbone after UCNP uptake.

When immature DCs encounter activation stimuli, they become activated to mature into immunostimulatory DCs. The maturation process results in upregulation of adhesion and costimulatory molecules and downregulation of the endocytic activity, and provides an optimal window for loading exogenous antigens on MHC class II molecules [32–34]. In order to further characterize the DC maturation induced by Pam3CSK4, the expression of CD86 on the surface of DC cells was examined by flow cytometry. When co-cultured for 24 h with different concentrations of UCNPs (0–25 µg/mL) surface-modified with Pam3CSK4, the expression level of CD86 was significantly increased (Fig. 5b), while UCNP/PAA/PEG and UCNP/PAA/PEG/OVA24 did not lead to increased expression of CD86. The data proves that surface-modified Pam3CSK4 could efficiently mature DCs. Our results show that with the help of UCNPs, the model antigen OVA24 could be effectively delivered to DCs, and Pam3CSK4 could lead to DC maturation and enhanced release of immunostimulatory cytokines.

### *In vitro* antigen cross-presentation

**Fig. 6:**
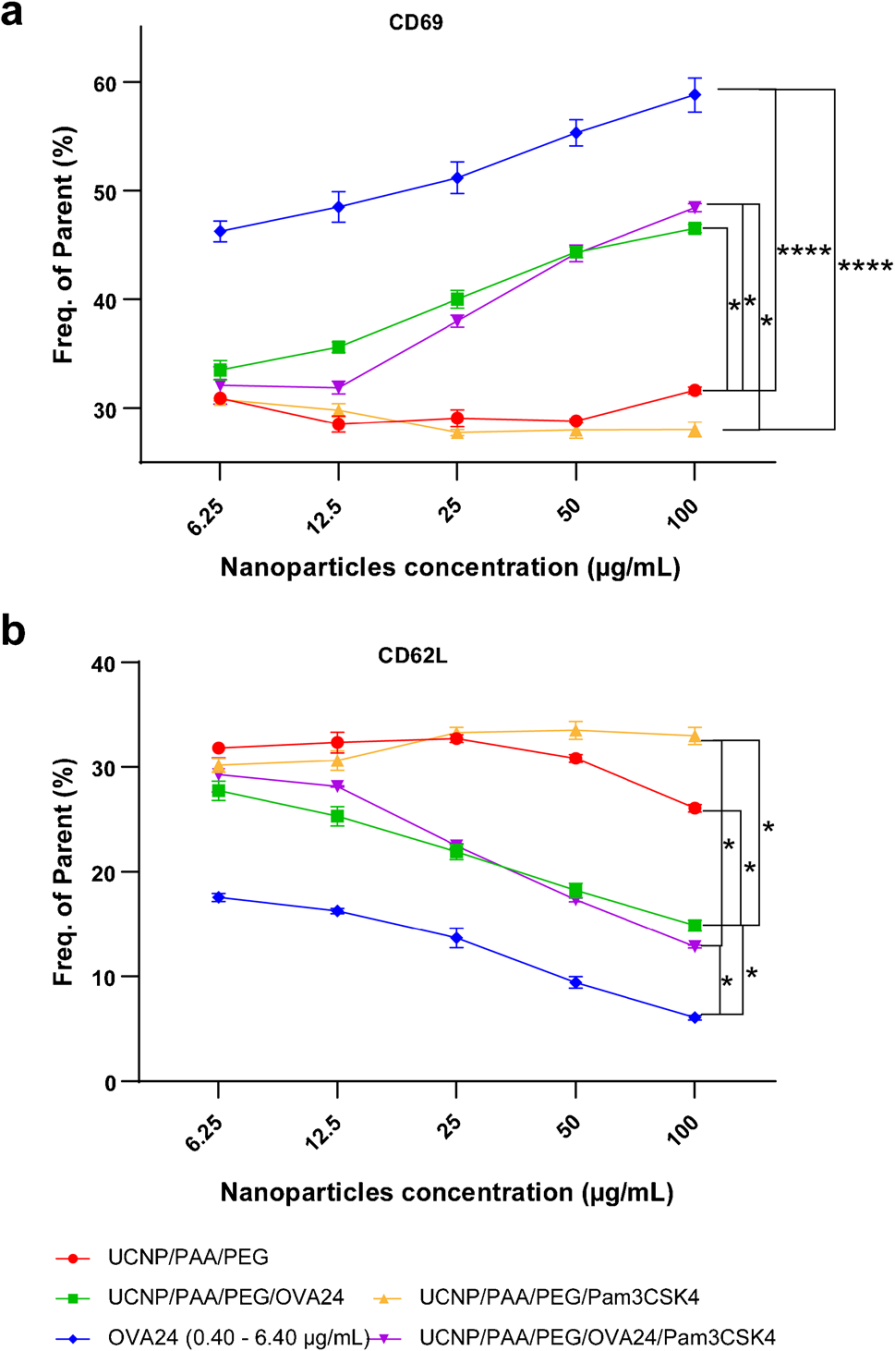
*In vitro* antigen presentation by DCs. OT-1 cells were assessed after co-culture with D1 DCs that were pulsed with different concentrations (6.25–100 µg/mL) of UCNPs. After 24 h of co-culture, the cells were harvested and OT-1 cells were stained with anti-CD69 (**a**) and anti-CD62L antibodies (**b**). The percentage of CD69-positive and CD62L-positive cells (within the population of CD8α-positive T cells) was measured by flow cytometry. Statistical significance was calculated using one-way ANOVA (*p < 0.05, ****p < 0.0001)

Next to DC maturation and activation, to raise an immune response requires efficient antigen presentation by DCs. Antigen presentation of the model antigen OVA can be determined by measuring the proliferation and activation of SIINFEKL-specific CD8^+^ T cells, commonly referred to as “OT-1 T cells” from transgenic mice that have MHC class I-restricted, OVA-specific CD8^+^ T cells [35]. To this end, T cells from the OT-1 TCR transgenic line were isolated to test their ability to recognize SIINFEKL peptide presented by DCs co-cultured with different formulations of UCNPs. T cell activation can be measured by assessing T cell proliferation, cytokine production, expression of activation markers, or downregulation of markers expressed on naive T cells [36]. Here, we focused on the expression of the T cell activation marker CD69 and the naïve T cell marker CD62L, a L-selectin, which after activation of T lymphocytes is rapidly downregulated. First, DCs were incubated with different concentrations of UCNPs (6.25–100 µg/mL) for 24 h, and subsequently co-cultured with OT-1 T cells. After 24 h, the expression of CD69 and CD62L on OT-1 T cells was assessed by flow cytometry (Fig. 6a–b). Our results demonstrate upregulation of the activation marker CD69 when DCs were loaded with UCNP surface-modified with OVA, or equal amounts of soluble OVA. The percentage of CD69-positive cells was slightly higher when DCs were pulsed with soluble OVA. As expected, in the absence of antigen, no changes in CD69 expression levels were observed when the cells were incubated with UCNP/PAA/PEG or UCNP/PAA/PEG/Pam3CSK4 (Fig. 6a). Similarly, when DCs were incubated with UCNP/PAA/PEG/OVA24 or UCNP/PAA/PEG/OVA24/Pam3CSK4, we observed downregulation of the naïve T cell marker CD62L (Fig. 6b). No changes in CD62L expression levels were observed when the cells were incubated with UCNP/PAA/PEG/Pam3CSK4. High concentrations of UCNP/PAA/PEG (100 µg/mL) slightly reduced expression of CD62L, indicating unspecific activation of immune cells. Even though surface-linked OVA was less efficient than soluble OVA, a robust activation of T cells could be observed *in vitro*. *In vitro*, DC can rapidly process and deliver soluble OVA, leading to specific T cell activation and increased CD69 expression, while surface-bound OVA requires gradual release from the conjugate to allow DC antigen presentation. As a consequence, soluble OVA is more effective at short time *in vitro*. *In vivo*, on the other hand, soluble OVA is rapidly degraded and eliminated by the immune system, resulting in less efficient T cell activation, while surface-bound OVA is protected from rapid cellular degradation and therefore increases the efficiency of T cell activation. Thus, surface-bound OVA may be more effective at longer time points *in vivo* and *in vitro* [37]. In summary, our results indicate that UCNP/PAA/PEG/OVA24 and UCNP/PAA/PEG/OVA24/Pam3CSK4 led to efficient OVA antigen processing and cross-presentation by DCs, followed by activation of OVA-specific T cells.

### *In vivo* antigen cross-presentation

**Fig. 7:**
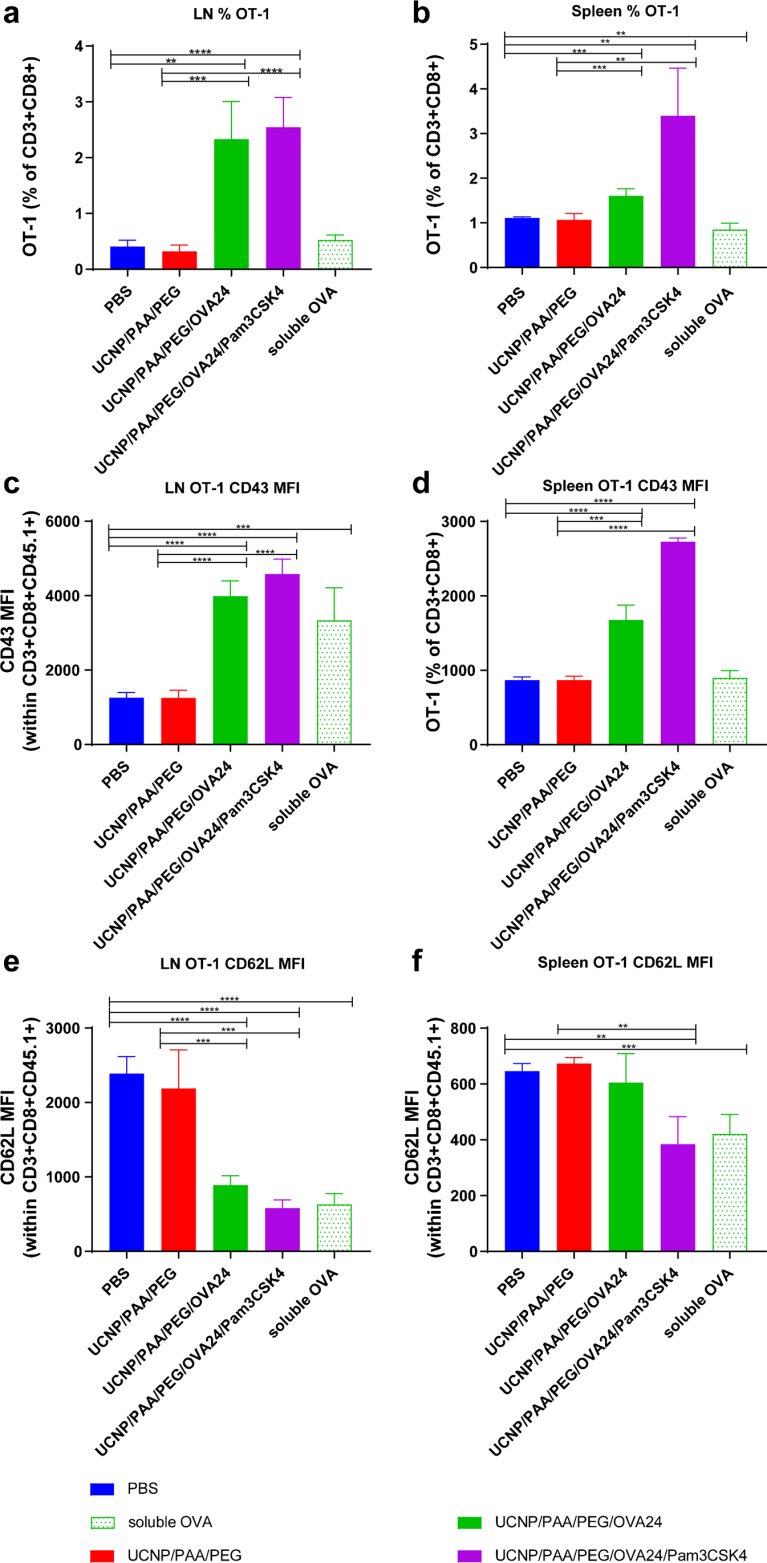
*In vivo* antigen presentation to CD8 T cells. Three hundred micrograms of UCNPs harboring 28 µg of OVA24 was injected subcutaneously into C57BL/6 mice. One day later, OT-1 cells (expressing the congenic CD45.1 marker) were transferred into these mice by intravenous injection. OT-1 cells were analyzed by flow cytometry with marker CD3^+^/CD8^+^ (**a**, **b**), marker CD43 (**c**, **d**), and CD62L antibody (**e**, **f**) in spleen and lymph node cell suspension at endpoint. Statistical significance was calculated using *t* test (**p < 0.01, ***p < 0.001, and ****p < 0.0001)

Next, we evaluated the potential of the UCNP formulations to induce antigen processing and cross-presentation of the OVA antigen *in vivo*. To test this hypothesis, UCNP surface-modified with OVA24 and/or Pam3CSK4, control NPs (UCNP/PAA/PEG), or soluble OVA were injected subcutaneously into C57BL/6 mice. One day post NP injection, 5 × 10^5^ OT-1 cells were adoptively transferred into these mice by intravenous injection. Transferring of these high numbers of OT-1 T cells increases the precursor frequency of OVA-specific T cells to artificially high levels, which allows us to detect subtle effects in cross-presentation of OVA antigen. Five days after injection, the spleen and tail-base draining inguinal lymph nodes were excised and the activation status and proliferation of transferred OVA-specific OT-1 cells were analyzed by flow cytometry. A strong expansion of OT-1 cells was found in the lymph nodes and spleen of mice treated with OVA-containing UCNPs, which proved superior to the administration of soluble OVA (Fig. 7a–b). Similarly, we observed the upregulation of the marker CD43, which is upregulated upon antigen-specific activation of T cells, in lymph nodes and spleens of mice treated with OVA-harboring UCNPs (Fig. 7c–d). At the same time, we observed downregulation of the naïve T cell marker CD62L (Fig. 7e–f). The absence of T cell proliferation and activation after injection of equal amounts of control UCNPs (UCNP/PAA/PEG), similar to the levels observed after PBS injection, showed that the UCNPs alone did not play a role in the expansion of T cells, and emphasize the antigen-specific nature of T cell activation. These results demonstrate the superior ability of OVA UCNPs over soluble OVA to induce antigen cross-presentation to OVA-specific CD8 T cells *in vivo*, and that the synergistic presence of OVA24 and Pam3CSK4 significantly increased the antigen-presenting ability of OVA UCNPs. These results are in agreement with our previous study demonstrating that NPs co-delivering antigen and adjuvant are superior over soluble vaccine components and increased antigenicity and adjuvanticity by enhancing cross-presentation [38].

### *In vivo* tracking of UCNPs

**Fig. 8:**
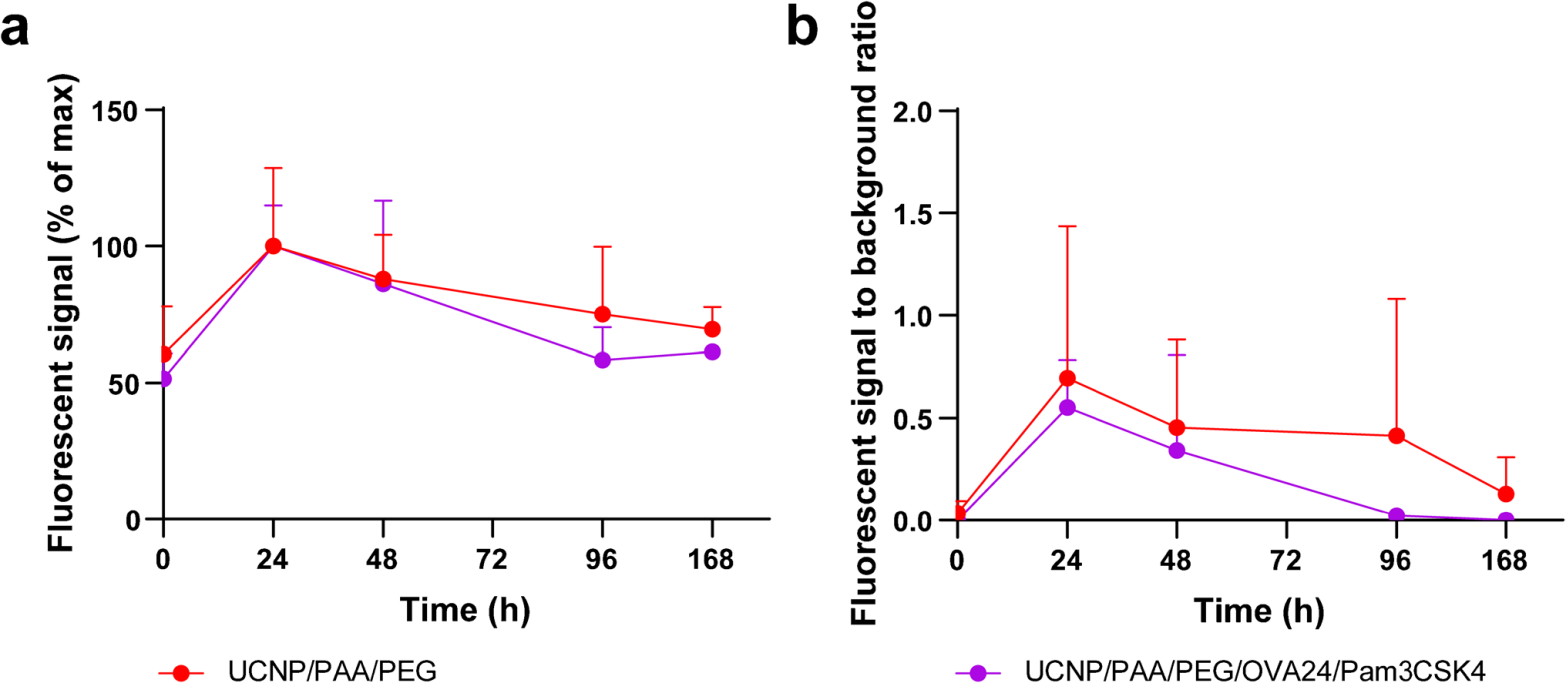
*In vivo* tracking of UCNP/PAA/PEG and UCNP/PAA/PEG/OVA24/Pam3CSK4. Under 980 nm laser excitation (600 mW/cm^2^), the fluorescent signal after of UCNPs was quantified at the draining (inguinal) lymph nodes 0 h, 24 h, 48 h, 96 h, and 168 h post-injection. **a** Fluorescent signal in draining lymph nodes is expressed as a percentage of maximum signal at 660 nm. **b** Fluorescent signal in the draining lymph nodes expressed as signal to background ratio at 660 nm. *N* = 4 mice per group

Several studies have shown that UCNPs can be used for highly sensitive stem cell and DC tracking *in vitro* and *in vivo* [39, 40]. Therefore, we evaluated whether UCNP/PAA/PEG/OVA24/Pam3CSK4 were multifunctional and could be used for DC immunotherapy and simultaneous *in vivo* monitoring of APCs after UCNP uptake (Figure S1). To this end, we injected UCNPs intradermally into the tail base and visualized the presence of UCNPs in the draining lymph nodes over time by molecular imaging. The tail-base injection site was chosen as it drains specifically to the inguinal lymph nodes which are located at sufficient distance in the mouse to be able to distinguish the injection site from the lymph nodes, and to visualize and quantify signals. To visualize the UCL of UCNPs in the lymph nodes *in vivo*, the interior platform of the animal housing unit of the IVIS Spectrum imager was adapted to hold a clamp which was attached onto a 980 nm laser head. The luminescence signal was collected by the IVIS internal emission filters at 660 nm. As shown in Fig. 8, within 24–48 h after injection, the fluorescence intensity in the lymph nodes gradually increased, and could be detected for a few days before it decreased almost to baseline levels 1 week after UCNP injection (Fig. 8a). After subtracting of the background signal quantified in the PBS group, the results show that at 24 h post-injection, UCL signal could be collected in the lymph nodes. Potentially, this may reflect the time that UCNP-pulsed and matured DCs need to migrate and to accumulate to the lymph nodes after stimulation with UCNP/PAA/PEG/OVA24/Pam3CSK4 and UCNP/PAA/PEG. The presence of OVA24 and Pam3CSK4 on the UCNP surface did not affect the kinetics of luminescence signal in the lymph nodes.

By comparing recent UCNP antigen presentation systems (Table S2), we have abandoned the unstable method of electrostatic adsorption and the oversized silica coating, chosen covalent coupling, which allows the antigen to be more firmly anchored to the UCNP and less susceptible to the microenvironment and reduces antigen shedding during transportation. In addition, the core–shell structure of UCNP we used exhibited a higher luminescence intensity and the presence of Pam3CSK4 successfully enhanced the DC activation efficiency. However, the luminescence intensity currently detected is still inadequate for clinical studies. The cucurbit[7]uril-based hydrophilic modification strategy has been shown to improve the luminescence efficiency of UCNP, and the possibility of integrating this strategy into our system would be a new direction for further research. Moreover, in our work, antibodies and agonist adjuvants are conjugated to UCNP via a carboxy-amino reaction, but UCNP often has multiple -COOHs on its surface, which can cause crosslinking reactions during the conjugation process resulting in poor conjugation efficiency. To avoid this phenomenon, a supramolecular self-assembly strategy is potentially an attractive tool to break this limitation.

## Conclusion

We have developed an UCNP platform that can effectively improve DC maturation and induce CD8^+^ T cell immune response. UCNP/PAA/PEG/OVA24/Pam3CSK4 did not only effectively deliver antigen, but also significantly improved the efficiency of DC activation and enhanced the release of cytokines compared with single antigen delivery nanoparticles (UCNP/PAA/PEG/OVA24). The excellent UCL efficiency of the UCNPs provided an effective means for monitoring UCNPs *in vivo*, reaching the highest value in murine lymph nodes 24 h after administration. Our UCNP platform provides a new strategy for cancer immunotherapy and simultaneous real-time *in vivo* imaging.

## Supplementary Information

Below is the link to the electronic supplementary material.

Supplementary file1 (DOCX 373 KB)
