# Supplementary material for: Upconversion nanoparticle platform for efficient dendritic cell antigen delivery and simultaneous tracking
Source: Mikrochim Acta. 2022 Sep 3;189(10):368. doi: 10.1007/s00604-022-05441-z (PMC9440881; doi:10.1007/s00604-022-05441-z)
Supplement: Supplementary file 1 — Supplementary file1 (DOCX 373 KB) [file 604_2022_5441_MOESM1_ESM.docx]

**Electronic Supplementary Material**

**Upconversion nanoparticle platform for efficient dendritic cell antigen delivery and simultaneous tracking**

Zhenfeng Yu^1^, Olena Vepris^1^, Christina Eich^1^*, Yansong Feng^2,3^, Ivo Que^1^, Marcel G.M. Camps^4^, Hong Zhang^2^, Ferry A. Ossendorp^4^, Luis J. Cruz^1^*

^1^Translational Nanobiomaterials and Imaging Group, Department of Radiology, Leiden University Medical Center, Albinusdreef 2, 2333 ZA Leiden, the Netherlands

^2^Van ‘t Hoff Institute for Molecular Sciences, University of Amsterdam, Science Park 904, 1098 XH Amsterdam, the Netherlands

^3^State Key Laboratory of Explosion Science and Technology, School of Mechatronical Engineering, Beijing Institute of Technology, Beijing 100081, China.

^4^Department of Immunology, Leiden University Medical Center, Albinusdreef 2, 2333 ZA Leiden, The Netherlands

Zhenfeng Yu, Olena Vepris, and Christina Eich shared first authorship.

* Corresponding authors’ email: Luis J. Cruz, L.J.Cruz_Ricondo@lumc.nl;

Christina Eich, C.Eich@lumc.nl

| Formulations | Zeta potential (mV) ± SD | PDI ± SD |
| --- | --- | --- |
| UCNP/PAA | -6.91 ± 0.07 | 0.15 ± 0.02 |
| UCNP/PAA/PEG | -21.3 ± 2.21 | 0.24 ± 0.02 |
| UCNP/PAA/PEG/OVA24 | -14.0 ± 1.52 | 0.34 ± 0.12 |
| UCNP/PAA/PEG/Pam3CSK4 | -14.6 ± 1.37 | 0.28 ± 0.12 |
| UCNP/PAA/PEG/OVA24/Pam3CSK4 | -13.8 ± 1.30 | 0.70 ± 0.04 |

**Table S1** Physicochemical properties of UCNP before and after functionalization with OVA24 and Pam3CSK4. The data are presented as mean ± SD (N=3).

**
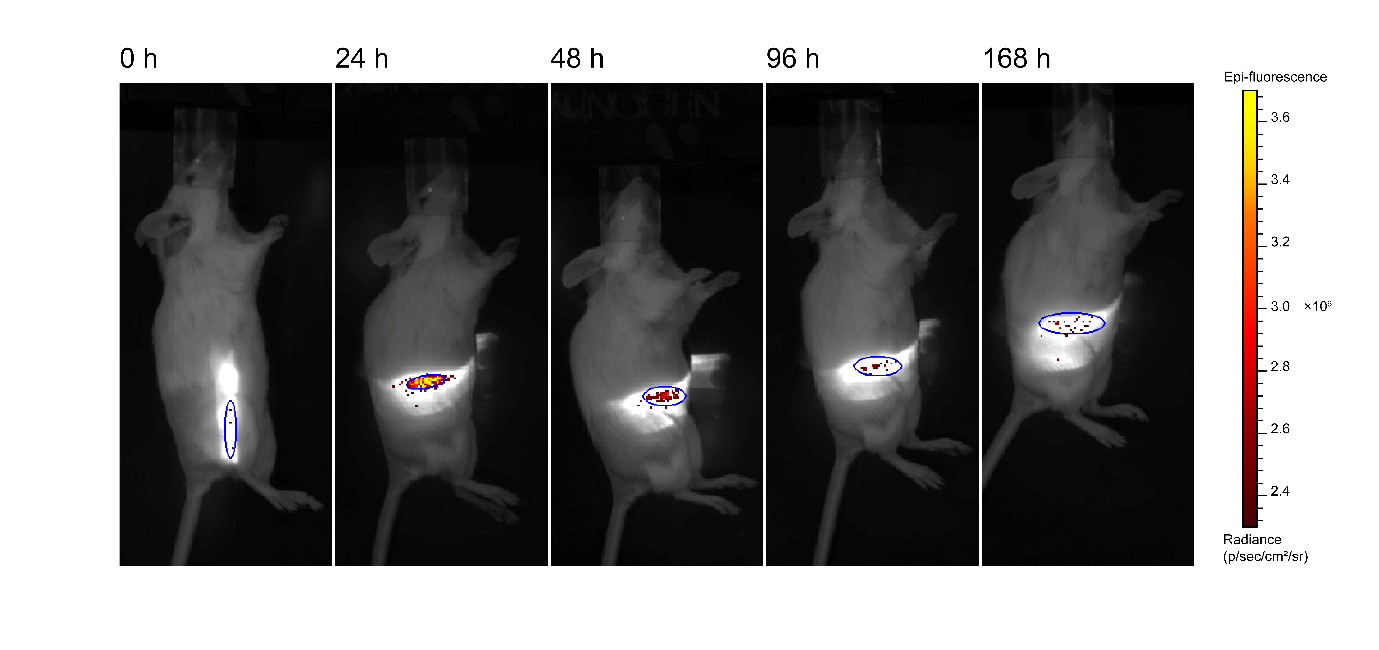
Figure S1** *In vivo* visualization of UCNP/PAA/PEG/OVA24/Pam3CSK4 at the right inguinal draining lymph nodes.

| **Base materials** | **Antigens or adjuvants** | **Formulation** | **Target** | **Reference** |
| --- | --- | --- | --- | --- |
| NaY/GdF_4_: YbEr | OVA | Electrostatic interaction | DCs | [40] |
| β-NaYF_4_: 20% Yb, 2% Er coated mesoporous silica | OVA/tumor cell fragment (TF) | Encapsulation | Tumor | [1] |
| NaYF_4_ :Tm | OVA | Conjugation | Melanoma | [2] |
| NaYF_4_: 2% Er, 20% Yb@ NaYF_4_ | OVA24, Pam3CSK4 | Conjugation | DCs |  |

**Table S2** An overview on recently OVA engineered UCNPs in cancer immunotherapy.

**References**

[1] Ding, B., Shao, S., Yu, C., Teng, B., Wang, M., Cheng, Z., Wong, K., Ma, P, Lin, J. (2018). Large‐pore mesoporous‐silica‐coated upconversion nanoparticles as multifunctional immunoadjuvants with ultrahigh photosensitizer and antigen loading efficiency for improved cancer photodynamic immunotherapy. Advanced Materials 30(52), 1802479. <https://doi.org/10.1002/adma.201802479>

[2] Lv, F., Jin, Y., Feng, X., Fan, M., Ren, C., Dai, X., ... & Liu, H. (2021). Traceable metallic antigen release for enhanced cancer immunotherapy. Journal of Nanoparticle Research 23(6): 1-11. <https://doi.org/10.1007/s11051-021-05256-8>
